# Impact of sugammadex on postoperative pneumonia and COPD exacerbations in COPD patients: A retrospective cohort study

**DOI:** 10.1097/MD.0000000000050293

**Published:** 2026-08-21

**Authors:** Fu-Chi Kang, Ting-Sian Yu, Chih-Wei Hsu, Yi-Chen Lai, Shu-Wei Liao, Kuo-Chuan Hung

**Affiliations:** a Department of Anesthesiology, Chi Mei Hospital, Chiali, Tainan, Taiwan; b Department of Anesthesiology, E-Da Hospital, I-Shou University, Kaohsiung, Taiwan; c Department of Psychiatry, Kaohsiung Chang Gung Memorial Hospital and Chang Gung University College of Medicine, Kaohsiung, Taiwan; d Department of Anesthesiology, Chi Mei Medical Center, Tainan, Taiwan.

**Keywords:** chronic obstructive pulmonary disease, neostigmine, neuromuscular blockade reversal, perioperative outcomes, postoperative pneumonia, sugammadex

## Abstract

Patients with chronic obstructive pulmonary disease (COPD) have an elevated risk of postoperative pulmonary complications. This study investigated whether sugammadex use was associated with a reduced risk of postoperative pneumonia and COPD exacerbations compared to neostigmine in this high-risk population. This retrospective cohort study utilized the TriNetX database to identify COPD patients undergoing elective surgery under general anesthesia between 2015 and 2024. Patients receiving rocuronium with sugammadex reversal were compared to those receiving neostigmine reversal using 1:1 propensity score matching. The primary outcome was 30-day postoperative pneumonia. Secondary outcomes included risks of COPD exacerbations, mortality, and intensive care unit (ICU) admission. After matching, 34,031 patients were analyzed per group. Sugammadex was associated with a reduced 30-day pneumonia incidence (1.8% vs 2.1%; hazard ratio [HR] 0.84, 95% confidence interval [CI] 0.75–0.94, *P* = .002) but increased ICU admissions (2.3% vs 1.9%; HR 1.22, 95% CI 1.10–1.36, *P* < .001). No significant differences were observed in COPD exacerbations (1.1% vs 1.3%; HR 0.90, 95% CI 0.79–1.04, *P* = .146) or mortality (0.89% vs 0.79%; HR 1.13, 95% CI 0.96–1.33, *P* = .147). Sensitivity and subgroup analyses revealed that benefits were more pronounced in academic centers (HR 0.64, 95% CI 0.57–0.73, *P* < .001) and elderly patients (HR 0.76, 95% CI 0.68–0.84, *P* < .001). Effects disappeared during the 30 to 90 day follow-up. Sugammadex use in COPD patients was associated with reduced 30-day postoperative pneumonia, particularly in elderly patients and academic settings. However, this benefit did not translate to fewer COPD exacerbations and was temporally limited. The increased ICU admissions likely reflect selection bias rather than causation.

## 1. Introduction

Chronic obstructive pulmonary disease (COPD) represents a significant global health burden, affecting over 300 million individuals worldwide and ranking as the third leading cause of mortality.^[1–3]^ Patients with COPD face substantially elevated risks when undergoing surgical procedures, particularly those requiring general anesthesia.^[4–6]^ The compromised respiratory function inherent to COPD predisposes these patients to postoperative pulmonary complications (PPCs), including pneumonia, respiratory failure, and disease exacerbation.^[7–9]^ These complications not only increase morbidity and mortality but also extend hospital stays and healthcare costs. Understanding how perioperative management choices influence respiratory outcomes in this vulnerable population remains crucial for improving surgical safety and optimizing patient care.

Neuromuscular blocking agents are routinely used during general anesthesia to facilitate intubation and surgical conditions.^[10,11]^ However, residual neuromuscular blockade following surgery can impair respiratory muscle function, compromise airway protective reflexes, and increase aspiration risk^[12,13]^ (concerns particularly relevant for COPD patients with preexisting respiratory compromise). Traditionally, neostigmine, an acetylcholinesterase inhibitor, has served as a standard reversal agent for neuromuscular blockade.^[14]^ Although effective, neostigmine has limitations, including incomplete reversal, particularly with profound blockade, and potential adverse effects through muscarinic stimulation, which may increase bronchial secretions and airway resistance.^[15,16]^ Sugammadex, a modified gamma-cyclodextrin, offers an alternative reversal mechanism by directly encapsulating rocuronium molecules.^[17,18]^ This novel approach provides a more rapid and complete reversal without cholinergic side effects,^[19–21]^ theoretically offering advantages for patients with compromised respiratory function.

Patients with COPD are particularly vulnerable to postoperative pneumonia owing to impaired mucociliary clearance, chronic airway inflammation, and reduced cough effectiveness.^[22,23]^ Importantly, pneumonia itself can precipitate acute COPD exacerbations through increased inflammatory mediators, mucus hypersecretion, and worsening airflow obstruction, creating a dangerous cycle of respiratory compromise.^[24]^ Despite this well-established relationship, limited research has specifically examined how the choice of neuromuscular reversal agent influences these respiratory outcomes in COPD patients. Therefore, this study aimed to investigate whether sugammadex, compared to neostigmine, reduces the incidence of postoperative pneumonia in this high-risk population. Additionally, we examined whether any reduction in pneumonia translates to decreased COPD exacerbations, potentially offering a strategy to improve both immediate and downstream respiratory outcomes in surgical patients with COPD.

## 2. Methods

### 2.1. Data sources

This retrospective cohort study used the TriNetX Research Network, a global federated health research platform that provides access to real-world electronic health records. The database predominantly encompasses healthcare organizations from the United States, containing comprehensive clinical information, including demographics, diagnoses, procedures, medications, laboratory results, and vital signs from over 120 million patients across more than 80 healthcare organizations. The platform maintains de-identified patient records in compliance with the Health Insurance Portability and Accountability Act, enabling large-scale epidemiological research while protecting patient privacy. Multiple peer-reviewed investigations have established TriNetX as a robust platform for conducting retrospective analyses and evaluating clinical outcomes across diverse patient populations.^[25–27]^ The Chi Mei Medical Center Institutional Review Board reviewed and approved this study protocol (IRB number: 11403-E03), waiving the requirement for informed consent given the retrospective design and utilization of anonymized patient data, which presented negligible privacy risks.

### 2.2. Study population and eligibility criteria

We identified adult patients (≥ 18 years) with a documented history of COPD who underwent elective surgical procedures under general anesthesia between January 1, 2015, and December 31, 2024. Eligible procedures included gastrointestinal, urological, and orthopedic surgeries. Patients were stratified into 2 cohorts based on the neuromuscular blockade reversal strategy employed during surgery. The sugammadex group consisted of patients who received rocuronium for muscle relaxation followed by sugammadex reversal, whereas the control group included those who received rocuronium with subsequent neostigmine reversal. The surgical date served as the index date for all participants.

Patients with documented stage 4 or 5 chronic kidney disease or those requiring dialysis before surgery were excluded, given the potential restrictions on sugammadex administration for severe renal impairment. We further excluded patients who developed acute kidney injury, respiratory failure, a history of intensive care unit (ICU) admission, or contracted coronavirus disease 2019 during the month preceding the surgery.

### 2.3. Data collection and matching strategy

Baseline demographic information and clinical characteristics were extracted from the 2-year period preceding the index date to comprehensively assess each patient’s health status. We employed propensity score matching to minimize selection bias and ensure comparable groups. Using a 1:1 greedy nearest-neighbor matching algorithm with a caliper width of 0.1 standard deviations of the logit of the propensity score, we paired patients based on key demographic factors (age, sex, race, and body mass index) and relevant comorbidities. Recognizing the importance of COPD severity in influencing outcomes, we specifically included COPD exacerbation history and inhaled corticosteroid use as matching variables to account for differences in disease severity between the groups.

### 2.4. Study outcomes

The primary endpoint was the development of pneumonia within 30 days of surgery. The secondary endpoints encompassed COPD exacerbation, all-cause mortality, and ICU admission during the same period. We included mortality and ICU admission as secondary outcomes to evaluate whether sugammadex might be preferentially administered to patients with greater disease severity, thereby assessing potential selection bias. To examine temporal patterns and detect delayed effects, we extended our analysis to capture these outcomes at 30 to 90 days following surgery.

### 2.5. Sensitivity analysis and subgroup analysis

We performed a sensitivity analysis to validate our findings’ robustness and address potential biases. This analysis restricted the cohort to patients treated exclusively at academic medical centers, where standardized protocols and consistent perioperative management practices might reduce variability in care delivery. This approach tested whether our observations persisted in a more uniform healthcare environment.

Subgroup analyses were used to examine the differential treatment effects across demographic strata. We analyzed outcomes separately by sex and age categories (18–65 years vs > 65 years) to identify potential effect modifications and assess whether sugammadex’s impact varied across these clinically relevant subgroups.

### 2.6. Statistical analysis

Descriptive statistics were used to summarize patient characteristics, with continuous data reported as means and standard deviations and categorical data as frequencies with percentages. No imputation was performed for missing data, and analyses were conducted using the data available within the TriNetX platform. We evaluated matching effectiveness by confirming that standardized mean differences remained below 0.1 across all variables and examining propensity score distribution overlap between groups. Survival analysis techniques addressed time-dependent outcomes, with Kaplan–Meier curves illustrating the cumulative incidence over time. Group comparisons utilized log-rank testing to detect differences in survival distribution. We calculated hazard ratios (HRs) with 95% confidence intervals (CIs) using Cox proportional hazards models, appropriately managing censored observations and accounting for varying follow-up periods. The analysis employed TriNetX’s integrated statistical tools, with statistical significance defined as a 2-tailed *P* value below .05, for all comparisons.

## 3. Results

### 3.1. Patient selection and baseline characteristics before and after matching

**Figure 1.**
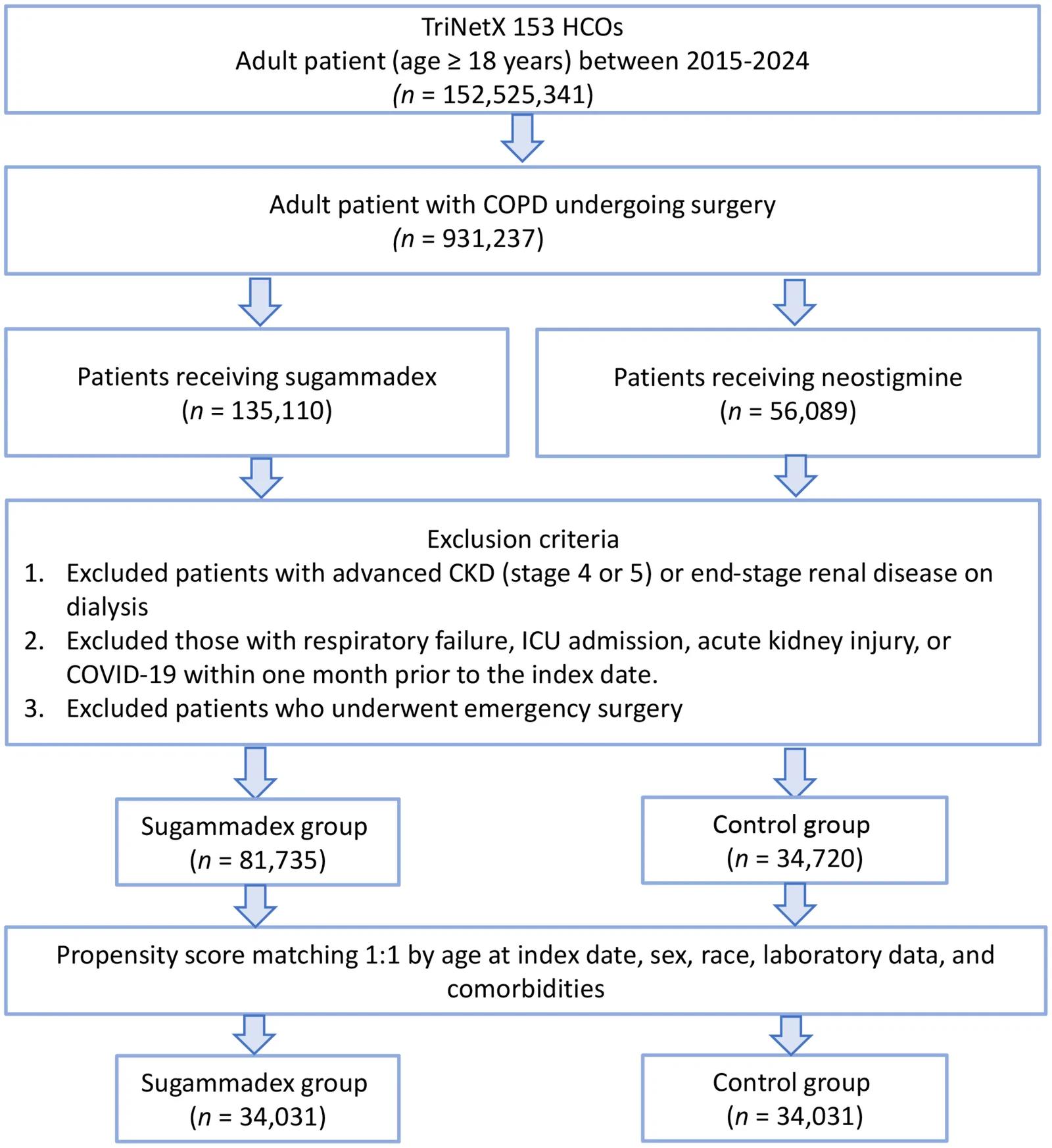
Patient selection flowchart for the retrospective cohort study using the TriNetX database. CKD = chronic kidney disease, COPD = chronic obstructive pulmonary disease, COVID-19 = coronavirus disease 2019, HCO = healthcare organization, ICU = intensive care unit, n = number of patients.

**Figure 2.**
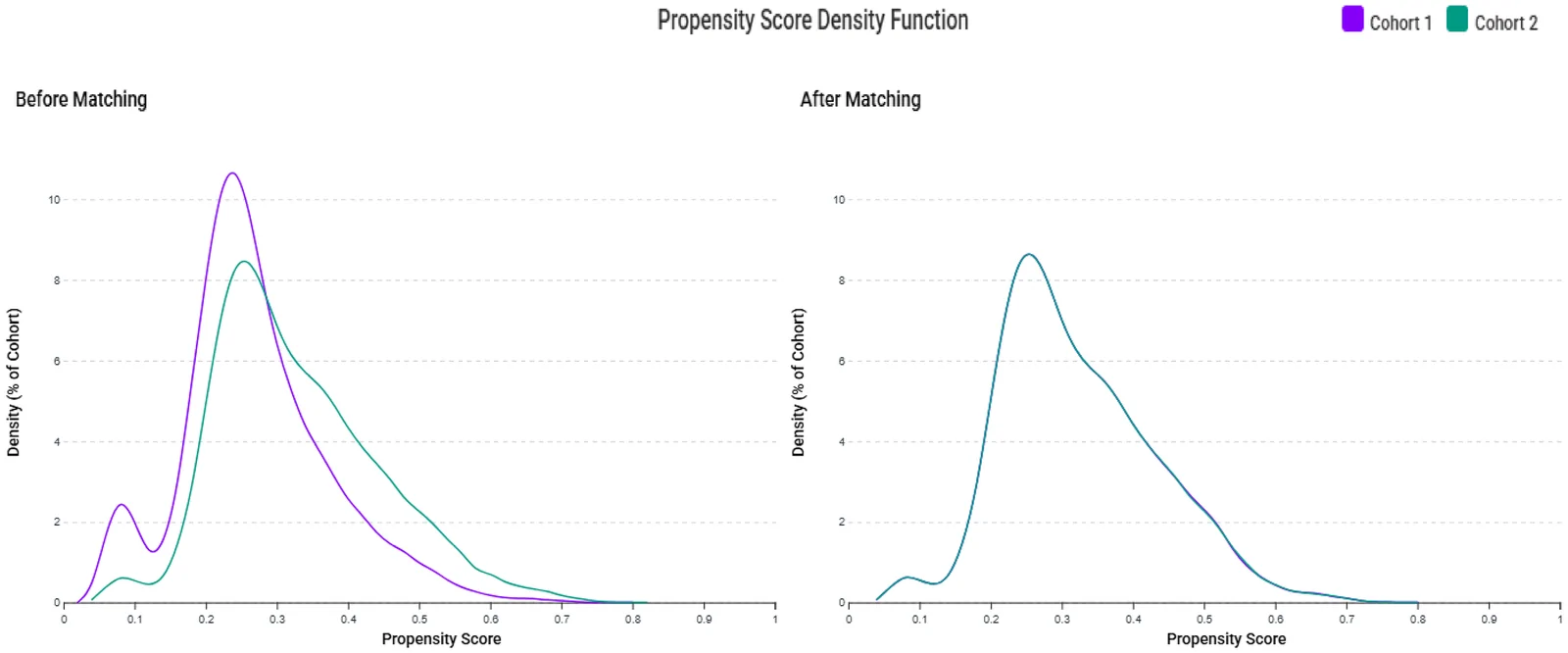
Propensity score density distributions before and after matching. The left panel shows distinct distribution patterns between the sugammadex group (Cohort 1) and the control group (Cohort 2) prior to matching. The right panel demonstrates the enhanced overlap and balance between groups following 1:1 propensity score matching using a caliper width of 0.1 standard deviations of the logit of the propensity score.

Initially, we identified 81,735 patients who received sugammadex for neuromuscular blockade reversal and 34,720 patients who received neostigmine as controls (Fig. 1). To ensure comparability between the groups and minimize selection bias, we employed 1:1 propensity score matching, resulting in 34,031 well-matched patients in each cohort. The propensity score matching process achieved an excellent balance between groups, as demonstrated by the density distributions before and after matching (Fig. 2). Prior to matching, substantial differences existed in the propensity score distributions between cohorts, but after matching with a caliper of 0.1 standard deviations, the distributions showed nearly complete overlap, indicating substantial improvement in measured covariate balance between groups.

Baseline characteristics after matching revealed well-balanced cohorts with standardized mean differences below 0.1 for all variables, confirming adequate matching quality (Table 1). Both groups had similar mean ages (64.4 years), with approximately 48% female representation. The prevalence of major comorbidities, including hypertension (66%), diabetes mellitus (27%), and ischemic heart disease (30%), was comparable. Importantly, indicators of COPD severity were well matched between the groups, with 15.4% versus 15.8% having documented COPD exacerbations and 8.5% versus 8.8% using inhaled corticosteroids in the sugammadex and control groups, respectively. Laboratory parameters, including hemoglobin levels and albumin concentrations, showed no significant differences between the matched cohorts.

**Table 1:** Baseline characteristics of patients before and after propensity score matching.

Variables	Before matching			After matching			
	Sugammadex group (n = 81735)	Control group (n = 34720)	SMD†		Sugammadex group (n = 34031)	Control group (n = 34031)	SMD†
Patient characteristics							
Age at index (yrs)	66.1 ± 11.8	64.2 ± 12.2	0.161		64.4 ± 12.1	64.4 ± 12.2	< 0.001
Female, n (%)	40,694 (49.8)	16,393 (47.2)	0.051		16,224 (47.7)	16,238 (47.7)	0.001
BMI, kg/m^2^	29.9 ± 8.1	29.8 ± 7.8	0.013		29.8 ± 8.0	29.8 ± 7.8	0.003
White, n (%)	66,267 (81.1)	25,866 (74.5)	0.159		25,863 (76.0)	25,724 (75.6)	0.010
Black or African American, n (%)	7293 (8.9)	3311 (9.5)	0.021		3239 (9.5)	3272 (9.6)	0.003
Other Race, n (%)	1635 (2.0)	512 (1.5)	0.040		501 (1.5)	512 (1.5)	0.003
Asian, n (%)	1188 (1.5)	368 (1.1)	0.035		348 (1.0)	368 (1.1)	0.006
Factors influencing health status and contact with health services, n (%)	76,763 (93.9)	31,258 (90.0)	0.143		30,784 (90.5)	30,772 (90.4)	0.001
Comorbidities, n (%)							
Essential (primary) hypertension	59,401 (72.7)	22,916 (66.0)	0.145		22,496 (66.1)	22,664 (66.6)	0.010
Neoplasms	44,340 (54.2)	16,281 (46.9)	0.148		16,157 (47.5)	16,128 (47.4)	0.002
Nicotine dependence	37,752 (46.2)	14,537 (41.9)	0.087		14,388 (42.3)	14,408 (42.3)	0.001
Anxiety, dissociative, stress-related, somatoform and other nonpsychotic mental disorders	34,083 (41.7)	12,012 (34.6)	0.147		11,802 (34.7)	11,916 (35.0)	0.007
Other anxiety disorders	30,193 (36.9)	10,515 (30.3)	0.141		10,302 (30.3)	10,426 (30.6)	0.008
Ischemic heart diseases	30,789 (37.7)	10,548 (30.4)	0.154		10,276 (30.2)	10,491 (30.8)	0.014
Diabetes mellitus	26,205 (32.1)	9459 (27.2)	0.106		9206 (27.1)	9379 (27.6)	0.011
Asthma	24,215 (29.6)	8942 (25.8)	0.087		8739 (25.7)	8851 (26.0)	0.008
Sleep apnea	24,882 (30.4)	8309 (23.9)	0.147		8081 (23.7)	8261 (24.3)	0.012
Diseases of liver	15,946 (19.5)	5468 (15.7)	0.099		5303 (15.6)	5419 (15.9)	0.009
COPD with exacerbation	16,731 (20.5)	5387 (15.5)	0.129		5236 (15.4)	5374 (15.8)	0.011
Cerebrovascular diseases	15,040 (18.4)	5024 (14.5)	0.106		4960 (14.6)	4988 (14.7)	0.002
Heart failure	15,264 (18.7)	4761 (13.7)	0.135		4681 (13.8)	4744 (13.9)	0.005
CKD	12,697 (15.5)	3762 (10.8)	0.139		3615 (10.6)	3749 (11.0)	0.013
Alcohol related disorders	9453 (11.6)	3416 (9.8)	0.056		3340 (9.8)	3397 (10.0)	0.006
Long term (current) use of inhaled steroids	10,732 (13.1)	2998 (8.6)	0.145		2905 (8.5)	2995 (8.8)	0.009
COVID-19	7274 (8.9)	870 (2.5)	0.278		915 (2.7)	870 (2.6)	0.008
Laboratory data, n (%)							
Hemoglobin > 12 g/dL	70,603 (86.4)	28,517 (82.1)	0.117		27,993 (82.3)	28,048 (82.4)	0.004
Hemoglobin A1c ≥ 7%	11,772 (14.4)	4544 (13.1)	0.038		4302 (12.6)	4451 (13.1)	0.013
Albumin g/dL (≥ 3.5 g/dL)	64,839 (79.3)	23,985 (69.1)	0.236		23,725 (69.7)	23,800 (69.9)	0.005

BMI = body mass index, CKD = chronic kidney disease, COPD = chronic obstructive pulmonary disease; COVID-19, coronavirus disease 2019, n = number of patients, SMD = standardized mean difference.

† An SMD value < 0.1 indicates adequate balance between groups.

### 3.2. Association between sugammadex and outcomes at 30-day and 30–90 day follow-up

Analysis of 30-day postoperative outcomes revealed significant differences between the treatment groups (Table 2). The incidence of pneumonia was significantly lower in patients receiving sugammadex than in those receiving neostigmine (1.8% vs 2.1%, HR 0.84, 95% CI 0.75–0.94, *P* = .002), representing a 16% relative risk reduction. However, this benefit was accompanied by an unexpected increase in ICU admissions among sugammadex recipients (2.3% vs 1.9%, HR 1.22, 95% CI 1.10–1.36, *P* < .001). COPD exacerbations showed a trend toward reduction with sugammadex but did not reach statistical significance (1.1% vs 1.3%, HR 0.90, 95% CI 0.79–1.04, *P* = .146), while mortality rates were similar between groups (0.89% vs 0.79%, *P* = .147).

**Table 2:** Association between sugammadex use and 30-day outcomes.

Outcomes	Sugammadex group (n = 34,031)	Control group (n = 34,031)	HR (95% CI)	*P* value
	Events (%)	Events (%)		
Pneumonia	603 (1.8)	717 (2.1)	0.84 (0.75–0.94)	.002
COPD exacerbation	391 (1.1)	433 (1.3)	0.90 (0.79–1.04)	.146
Mortality	302 (0.89)	268 (0.79)	1.13 (0.96–1.33)	.147
ICU admission	776 (2.3)	637 (1.9)	1.22 (1.10–1.36)	< .001

CI = confidence interval, COPD = chronic obstructive pulmonary disease, HR = hazard ratio, ICU = intensive care unit, n = number of patients.

An extended follow-up analysis examining the outcomes between 30 and 90 days postoperatively revealed a different pattern (Table 3). The protective effect of sugammadex against pneumonia observed in the immediate postoperative period was no longer evident (1.5% in both groups, HR 0.99, 95% CI 0.87–1.12, *P* = .816). Similarly, COPD exacerbation rates were identical between the groups (1.6% each, HR 1.02, 95% CI 0.90–1.15, *P* = .781), and no significant differences emerged in mortality or ICU admission rates during this extended period. These findings suggest that the benefits of sugammadex are primarily confined to the immediate postoperative period.

**Table 3:** Association between sugammadex use and 30 to 90 day outcomes.

Outcomes	Sugammadex group (n = 31,876)	Control group (n = 31,876)	HR (95% CI)	*P* value
	Events (%)	Events (%)		
Pneumonia	474 (1.5)	489 (1.5)	0.99 (0.87–1.12)	.816
COPD exacerbation	524 (1.6)	524 (1.6)	1.02 (0.90–1.15)	.781
Mortality	298 (0.94)	274 (0.86)	1.11 (0.94–1.30)	.226
ICU admission	297 (0.93)	276 (0.87)	1.09 (0.93–1.29)	.281

CI = confidence interval, COPD = chronic obstructive pulmonary disease, HR = hazard ratio, ICU = intensive care unit, n = number of patients.

### 3.3. Sensitivity analysis

To validate our findings and address potential variations in care quality, we performed a sensitivity analysis restricted to patients treated at academic medical centers (Table 4). This analysis included 21,826 matched pairs and demonstrated even more pronounced benefits of sugammadex. The reduction in pneumonia risk was substantially greater in this subset (1.9% vs 2.9%, HR 0.64, 95% CI 0.57–0.73, *P* < .001), representing a 36% relative risk reduction. Notably, the increased ICU admission rate observed in the primary analysis was not replicated in academic centers (3.1% vs 3.0%, HR 1.05, 95% CI 0.95–1.18, *P* = .334), suggesting that the association between sugammadex and ICU admission may be influenced by institutional factors or patient selection practices that vary between academic and nonacademic settings.

**Table 4:** Sensitivity analysis.

Outcomes	Sugammadex group (n = 21,826)	Control group (n = 21,826)	HR (95% CI)	*P* value
	Events (%)	Events (%)		
Pneumonia	405 (1.9)	628 (2.9)	0.64 (0.57–0.73)	< .001
COPD exacerbation	345 (1.6)	331 (1.5)	1.05 (0.90–1.22)	.546
Mortality	182 (0.83)	173 (0.79)	1.06 (0.86–1.31)	.577
ICU admission	676 (3.1)	643 (3.0)	1.05 (0.95–1.18)	.334

CI = confidence interval, COPD = chronic obstructive pulmonary disease, HR = hazard ratio, ICU = intensive care unit, n = number of patients.

### 3.4. Subgroup

Subgroup analyses revealed important heterogeneity in treatment effects across demographic categories. Sex-based analysis (Table 5) demonstrated consistent pneumonia reduction with sugammadex in both males (HR 0.81, 95% CI 0.70–0.93, *P* = .003) and females (HR 0.80, 95% CI 0.70–0.92, *P* = .001). However, an unexpected finding emerged regarding mortality, with females showing a significantly increased risk when receiving sugammadex (HR 1.43, 95% CI 1.11–1.84, *P* = .005), while males showed no such association. Both sexes experienced increased ICU admission rates with sugammadex, although this reached statistical significance only in females.

**Table 5:** Subgroup analysis based on sex.

Outcomes	Male (n = 15,592)		Female (n = 17,590)	
	HR (95% CI)	*P* value	HR (95% CI)	*P* value
Pneumonia	0.81 (0.70–0.93)	.003	0.80 (0.70–0.92)	.001
COPD exacerbation	1.00 (0.83–1.18)	.961	0.96 (0.82–1.13)	.629
Mortality	0.95 (0.75–1.20)	.659	1.43 (1.11–1.84)	.005
ICU admission	1.13 (1.00–1.28)	.054	1.22 (1.07–1.39)	.003

CI = confidence interval, COPD = chronic obstructive pulmonary disease, HR = hazard ratio, ICU = intensive care unit, n = number of patients.

Age-stratified analysis (Table 6) revealed that the pneumonia-protective effect of sugammadex was primarily driven by elderly patients. Among those over 65 years of age, sugammadex significantly reduced pneumonia risk (HR 0.76, 95% CI 0.68–0.84, *P* < .001), while younger patients showed no significant benefit (HR 0.88, 95% CI 0.73–1.06, *P* = .174). Conversely, younger patients experienced a significant reduction in COPD exacerbations with sugammadex (HR 0.75, 95% CI 0.60–0.94, *P* = .012), a benefit not observed in the elderly cohort. The increased ICU admission risk associated with sugammadex was consistent across age groups, suggesting that this finding is not age-dependent.

**Table 6:** Subgroup analysis by age.

Outcomes	18–65 years (n = 11,199 for each group)		>65 years (n = 24,990 for each group)	
	HR (95% CI)	*P* value	HR (95% CI)	*P* value
Pneumonia	0.88 (0.73–1.06)	.174	0.76 (0.68–0.84)	< .001
COPD exacerbation	0.75 (0.60–0.94)	.012	0.96 (0.84–1.09)	.489
Mortality	1.44 (0.92–2.26)	.110	1.11 (0.94–1.31)	.230
ICU admission	1.31 (1.10–1.55)	.002	1.18 (1.07–1.30)	.001

CI = confidence interval, COPD = chronic obstructive pulmonary disease, HR = hazard ratio, ICU = intensive care unit, n = number of patients.

## 4. Discussion

This large retrospective cohort study demonstrated that sugammadex use in COPD patients undergoing elective surgery was associated with a 16% reduction in 30-day postoperative pneumonia compared with neostigmine. This protective effect against pneumonia was more pronounced in academic medical centers (36% reduction) and primarily benefited elderly patients. While sugammadex appeared to be associated with increased ICU admissions in the overall analysis, this finding likely reflects selection bias (where clinicians preferentially chose sugammadex for higher-risk patients) rather than a causal relationship. This interpretation is supported by the absence of increased ICU admissions in the academic center subgroup, where standardized protocols presumably reduced such selection bias. Importantly, sugammadex showed no significant impact on COPD exacerbation or mortality. All observed differences between the treatment groups were confined to the immediate 30-day postoperative period, with no sustained effects detected during extended follow-up, suggesting that the benefits of sugammadex are temporally limited but clinically meaningful during the critical postoperative recovery phase.

Evidence regarding the impact of sugammadex on PPCs remains markedly inconsistent across published studies. Large matched cohort analyses and randomized trials in general surgical and thoracic populations have demonstrated that sugammadex may reduce major pulmonary events, including pneumonia and respiratory failure.^[19,21,28]^ These studies suggest that eliminating residual paralysis and avoiding cholinergic side effects could translate into meaningful clinical benefits. However, this protective effect has not been universally observed, with several well-designed studies reporting neutral or negative findings. Ko et al found no significant difference in PPC rates between sugammadex and neostigmine in patients undergoing spine surgery,^[29]^ while Lee et al reported similar null findings in lung surgery patients.^[30]^ More concerning, recent evidence from high-risk bronchoscopy patients suggested a paradoxically higher incidence of PPCs with sugammadex, although the absolute difference was small, and its clinical relevance remains uncertain.^[31]^ These conflicting results likely stem from multiple sources of heterogeneity, including variations in patient risk profiles, surgical procedures, institutional practices, and definitions of pulmonary complications. Most critically, the existing literature lacks robust data specifically examining COPD patients, who possess unique pathophysiological characteristics that may modify treatment responses differently than the general surgical population. This gap in evidence leaves considerable uncertainty regarding whether sugammadex offers true benefits or potential risks in this particularly vulnerable patient group.

Our finding of reduced pneumonia risk with sugammadex aligns with several previous studies,^[19,21,28]^ although the magnitude of the effect varies considerably across investigations. The 16% relative risk reduction observed in our primary analysis is more conservative than some reports but likely reflects real-world effectiveness across diverse healthcare settings. The sensitivity analysis restricted to academic medical centers revealed a more pronounced 36% risk reduction, suggesting that standardized perioperative protocols and specialized respiratory care may amplify the benefits of sugammadex. This finding underscores the importance of considering a broader perioperative management setting, as drug effectiveness may be strongly influenced by how it is integrated into overall care strategies. Subgroup analysis revealed that the pneumonia-protective effect was primarily concentrated in elderly patients (24% risk reduction in those aged > 65 years), whereas younger patients showed no significant benefit. This age-dependent response likely reflects the higher baseline pneumonia risk in elderly COPD patients, where even modest improvements in neuromuscular function recovery could meaningfully impact the outcomes.

Despite our hypothesis that reducing pneumonia would subsequently decrease COPD exacerbations, we observed no significant difference in exacerbation rates between the treatment groups. This finding challenges the presumed mechanistic connection between postoperative pneumonia and COPD exacerbations associated with the use of neuromuscular reversal agents. Several explanations merit consideration. First, COPD exacerbations have multifactorial triggers beyond pneumonia, including viral infections, environmental exposures, and medication nonadherence, which sugammadex cannot influence.^[32,33]^ Second, the relatively low overall exacerbation rate (approximately 1.2%) may have limited the statistical power to detect differences. Interestingly, younger patients showed reduced exacerbations with sugammadex, suggesting that age may modify the relationship between neuromuscular reversal and airway inflammation in ways that warrant further investigation.

The increased ICU admission rate associated with sugammadex in the primary analysis appears paradoxical, given the reduced incidence of pneumonia. However, this finding likely reflects a selection bias rather than a causal relationship. In real-world practice, anesthesiologists may preferentially choose sugammadex for patients perceived as at higher risk or for those developing intraoperative complications. This interpretation is supported by a sensitivity analysis from academic centers, where presumably more standardized protocols eliminated the association between sugammadex and ICU admission. The similar mortality rates between the groups, despite differences in ICU utilization, suggest that sugammadex may indeed benefit critically ill patients by preventing pneumonia in high-risk situations.

This study has several important limitations that must be considered when interpreting the results. First, the retrospective observational design precludes causal inference, and residual confounding remains possible despite propensity score matching. We could not account for intraoperative factors that might influence outcomes, such as depth of neuromuscular blockade, timing of reversal, or specific ventilation strategies. Second, reliance on diagnostic codes may underestimate the true pneumonia incidence, as mild cases might go undiagnosed or uncoded. Third, COPD severity assessment was limited to available variables, and spirometric data confirming the diagnosis and staging were unavailable. Fourth, the predominantly United States-based population may limit the generalizability to other healthcare systems with different perioperative practices. Finally, the loss of treatment effect beyond 30 days suggests that our findings, while clinically relevant for immediate postoperative care, should not be extrapolated to long-term outcomes.

## 5. Conclusion

In this large real-world study of COPD patients undergoing elective surgery, sugammadex use was linked to a lower 30-day postoperative pneumonia rate than neostigmine use, especially among elderly patients and in academic hospitals. However, this advantage did not translate into fewer COPD exacerbations and was associated with higher ICU admission rates, likely due to selection bias. The observed benefits were most pronounced in the immediate postoperative phase and in settings with standardized care, indicating that the effectiveness of sugammadex is influenced by both patient characteristics and institutional protocols. These results support the selective use of sugammadex in elderly COPD patients at high risk for pneumonia while underscoring the need for prospective studies to establish causality and refine its clinical application.

## Author contributions

**Conceptualization:** Fu-Chi Kang, Ting-Sian Yu, Chih-Wei Hsu, Kuo-Chuan Hung.

**Data curation:** Fu-Chi Kang, Ting-Sian Yu, Shu-Wei Liao, Kuo-Chuan Hung.

**Formal analysis:** Fu-Chi Kang, Yi-Chen Lai, Shu-Wei Liao, Kuo-Chuan Hung.

**Investigation:** Chih-Wei Hsu.

**Methodology:** Ting-Sian Yu, Chih-Wei Hsu, Yi-Chen Lai.

**Resources:** Ting-Sian Yu, Shu-Wei Liao.

**Software:** Yi-Chen Lai.

**Validation:** Chih-Wei Hsu, Yi-Chen Lai.

**Writing – original draft:** Fu-Chi Kang, Kuo-Chuan Hung.

**Writing – review & editing:** Fu-Chi Kang, Kuo-Chuan Hung.
